# Memory and Fitness Optimization of Bacteria under Fluctuating Environments

**DOI:** 10.1371/journal.pgen.1004556

**Published:** 2014-09-25

**Authors:** Guillaume Lambert, Edo Kussell

**Affiliations:** 1 The Institute of Genomics and Systems Biology, The University of Chicago, Chicago, Illinois, United States of America; 2 Department of Biology and Center for Genomics and Systems Biology, New York University, New York, New York, United States of America; 3 Department of Physics, New York University, New York, New York, United States of America; Université Paris Descartes, INSERM U1001, France

## Abstract

Bacteria prudently regulate their metabolic phenotypes by sensing the availability of specific nutrients, expressing the required genes for their metabolism, and repressing them after specific metabolites are depleted. It is unclear, however, how genetic networks maintain and transmit phenotypic states between generations under rapidly fluctuating environments. By subjecting bacteria to fluctuating carbon sources (glucose and lactose) using microfluidics, we discover two types of non-genetic memory in *Escherichia coli* and analyze their benefits. First, *phenotypic memory* conferred by transmission of stable intracellular *lac* proteins dramatically reduces lag phases under cyclical fluctuations with intermediate timescales (1–10 generations). Second, *response memory*, a hysteretic behavior in which gene expression persists after removal of its external inducer, enhances adaptation when environments fluctuate over short timescales (<1 generation). Using a mathematical model we analyze the benefits of memory across environmental fluctuation timescales. We show that memory mechanisms provide an important class of survival strategies in biology that improve long-term fitness under fluctuating environments. These results can be used to understand how organisms adapt to fluctuating levels of nutrients, antibiotics, and other environmental stresses.

## Author Summary

Bacterial adaptation to new environments typically involves reorganization of gene expression that temporarily decreases growth rates. By exposing cells to fluctuating conditions using an innovative microfluidic device, we discover that *E. coli* cells can remember past environments, which accelerates their physiological adaptation. Using a modeling approach combined with experiments, we demonstrate the adaptive advantage of memory for organisms that 1) transmit long-lived intracellular proteins between generations or 2) respond to fluctuations in a history-dependent manner. Our work describes one of the simplest examples of adaptive memory in a living organism and provides significant insights into the behavior of genetic networks under diverse fluctuations, including nutrients, antibiotics, and other environmental stresses.

## Introduction

*Escherichia coli* cells grown in the presence of both glucose and lactose first consume glucose, which is more easily metabolized, before expressing the genes necessary for lactose catabolism [1]–[3]. The prioritization of bacterial metabolism toward a specific substrate is achieved by catabolite repression and is often observed in microorganisms grown in the presence of multiple carbon sources [4]. Metabolite selection usually favors more accessible energy sources when multiple substrates are available [5]. Since transitions between metabolic phenotypes incur a significant growth rate cost, microorganisms are faced with a fitness optimization problem in temporally fluctuating environments [6]. For instance, a premature commitment to new metabolic substrates initially present in low quantities may limit long-term fitness if levels remain insufficient to support growth. Similarly, a delayed phenotypic switch may reduce overall nutrient intake and, as a result, cells may be outcompeted by populations with a more timely response.

Recently, simple laws that relate bacterial growth, translational efficiency, and metabolic rates have been revealed through a combination of theory and experiments [7]. These laws, which hold for bacteria growing in constant environments (e.g. in chemostats), can be used to predict key features of bacterial adaptation, including fitness landscapes of drug resistance [8]. In fluctuating environments, however, little quantitative data exists on the physiological strategies that bacteria use to optimize growth. When environments fluctuate, steady-state growth may not be achieved in any given environment, and long-term growth rates must be measured across multiple fluctuations over longer timescales. Experimentally, this presents challenges that are currently being addressed using microfluidics and microscopy, e.g. yeast have been grown in alternating sugars [9], while bacteria have been exposed to single step changes of carbon source [10]. Here, we present experiments in bacteria over longer timescales, during which many back-and-forth nutrient fluctuations are made while continuously measuring cellular growth. In particular, we probe gene regulatory networks using an innovative microfluidics device over timescales that have not previously been examined and discover that memory-based bacterial growth strategies constitute a primary mode of adaptation.

Memory in bacteria has been studied in the context of epigenetic switches [11], which can maintain stable phenotypic states over hundreds of generations. It was recently demonstrated that cell fate decisions in *Bacillus subtilis* employ memory in the transition between sessile chaining and motility [12]. More broadly, historical growth conditions are known to alter several bacterial responses [13], implying that memory may be present in multiple cellular processes. Indeed, the presence of feedback loops, coupled with a tuning of gene expression levels, can introduce hysteresis and memory in genetic networks [14].

Despite these observations, very little is known regarding how memory influences growth rates and, more importantly, the recovery from sudden environmental changes. Moreover, along with prudent gene regulation, bacteria employ an additional fail-safe known as the stringent response, which is useful in case carbon starvation persists and cell viability becomes compromised. Coordinated by the signaling molecule (p)ppGpp, which accumulates under either amino-acid depletion or carbon starvation [15]–[17], the resulting protective state exhibits growth arrest, lowered translational and metabolic activity [18], and expression of biosynthetic genes [19]. Cellular memory of historical phenotypes could impart a key advantage in a changing environment by alleviating the cost of frequent regulatory switches, as well as mitigating the stringent response, while allowing cells to adapt to multiple environments. This possibility, while beneficial in theory [20], has not been tested or quantified experimentally.

**Figure 1:**
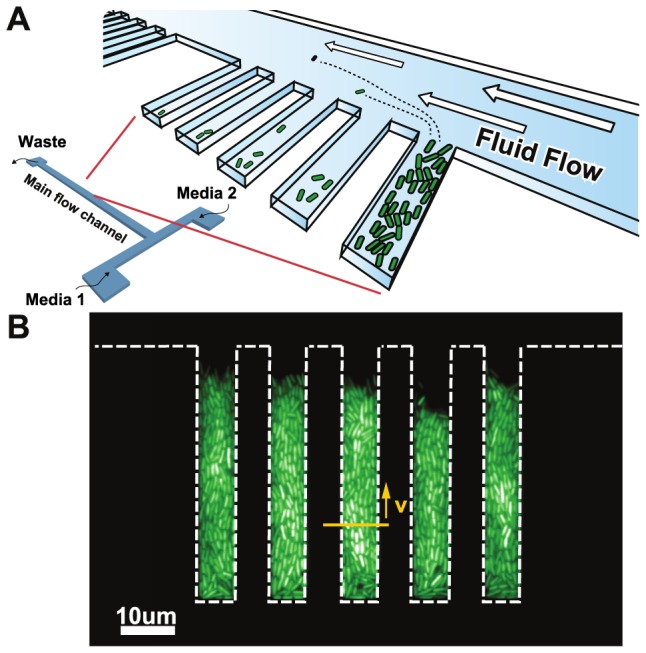
Chemoflux device for growth rate measurement in changing environments. A) Schematic representation of the microfluidic device. B) Fluorescence micrograph of cells grown inside the growth chambers (white dashed lines outline the growth chambers). The elongation rate of the cells is quantified using the lateral speed 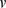 of cells 15 microns away from the closed end of the growth chamber (yellow arrow).

We investigate memory-based adaptive mechanisms by asking whether bacterial cells grown in fluctuating environments would either adopt a mixed phenotype and remember adaption to both environments, avoid metabolic switching altogether and lock into a single phenotype, or fail to fully adapt to either environment and remain in a partially adapted state. We developed a microfluidic device that maintains growing bacterial populations inside microscopic growth chambers (GCs) to study phenotypic changes that occur in *E. coli* in response to sudden environmental changes (see Fig. 1a for schematic representations of the device). Our microfluidic device shares certain properties with a chemostat – namely the maintenance of a constant population size and a steady influx of fresh nutrients. However, while a chemostat maintains a static chemical environment, the small volume of our device allows the chemical environment to be changed very rapidly (Fig. S1 and Text S1). Since the chemical environment is not stable but rather in constant flux, we call our device a *chemoflux*. Its design builds on previous ones used to study bacterial aging [21], yeast fitness under a changing environment [9], or to characterize growth of mycobacterial cells [22].

## Results

### Phenotypic memory in response to sudden environmental changes

**Figure 2:**
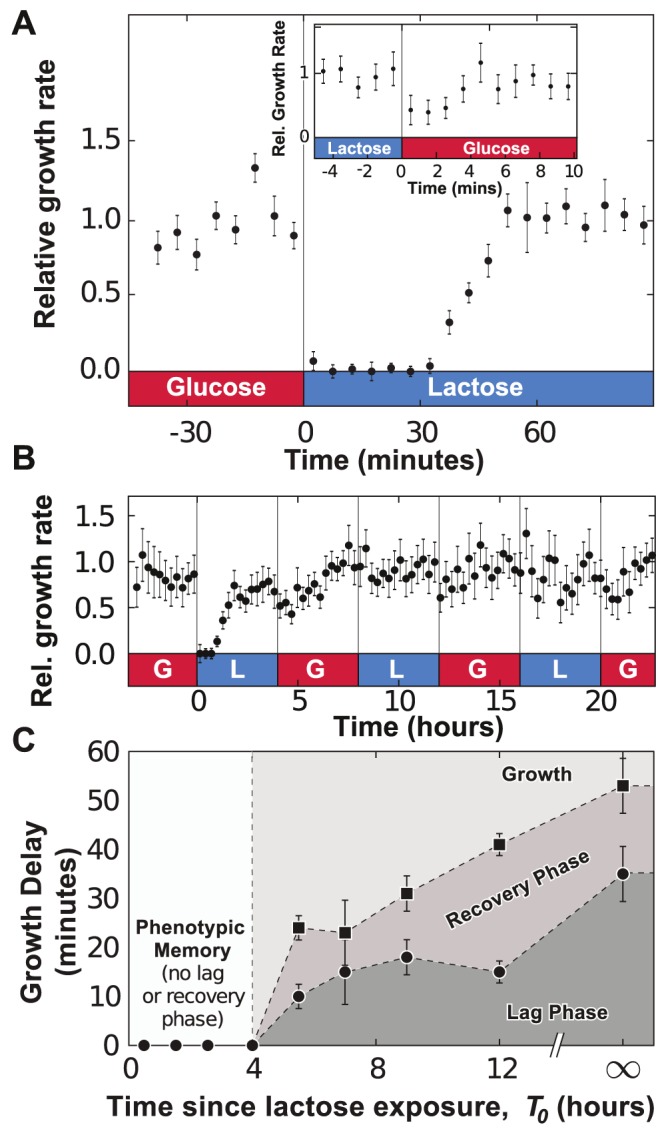
Adaptation to fluctuating environments. A) Following a glucose-to-lactose transition, the growth rate of the cells decreases to zero before progressively relaxing back to its equilibrium value in about 55 minutes. Data is binned over 5-minute intervals and error bars are computed from the standard error of the mean (SEM) with N = 5 GCs. Inset: A much less pronounced effect is observed during lactose-to-glucose transitions, where cell growth recovers fully after 5 minutes (no data binning). B) Under fluctuating conditions (environmental duration 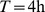, data binned over 15-minute intervals), cells remember previous *lac* induction and do not enter a lag phase when lactose is reintroduced. C) The duration of the lag (bullets) and lag+recovery phases (black squares) for *lac*-induced cells depends on the amount of time between lactose exposures. Error bars are computed from the standard error of the regression parameters used to measure the lag+recovery times.

The growth rate of *E. coli* cells is quantified using the lateral displacement of cells 10 to 15 microns away from the end of the growth chambers (Fig 1b, see Materials and Methods section for further details). As we changed the media flowing in the main channel from MOPS minimal media (MMM) supplemented with 0.4% glucose to MMM+0.4% lactose, a lag phase, manifested as vanishing lateral movement, occurred immediately following the environmental change (Fig. 2a and Supp. Video S2) and lasted approximately 35 minutes. Following the lag phase, cells entered a recovery phase and progressively resumed growth as the lateral speed reached a stable rate 55 minutes after the glucose-to-lactose transition. The measured durations of the lag+recovery phases in our device are in agreement with bulk measurements of diauxic shifts, where transitions between glucose- and lactose-consuming phenotypes occur within an hour on average [23]. On the other hand, lactose-to-glucose transitions do not impose a significant metabolic burden on the cells and cellular growth recovered within 5 minutes (Fig. 2a, inset). The determinants of lag and recovery phases are analyzed in the next section of Results.

To investigate whether *lac* induction is remembered after the removal of lactose, we monitored the growth dynamics of cells in an environment where MMM+0.4% glucose and MMM+0.4% lactose conditions alternate every 4 hours (here, we define 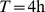 as the environmental duration). In Fig. 2b, cells were subjected to three consecutive glucose/lactose cycles: the first glucose-to-lactose transition resulted in significant lag+recovery phases, but cells were able to grow on lactose without having to go through a lag phase when we reintroduced lactose at 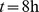 and 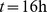. The response to glucose/lactose transitions eventually became seamless, indicating that cells conserved the ability to metabolize lactose through the 4-hour exposures to glucose. Cells grown under cyclical glucose/lactose conditions thus displayed *phenotypic memory* of their previous metabolic adaptation.

We determined the timescale over which phenotypic memory persists by analyzing the dependence of the lag phase on the time since the last exposure to lactose. Fully induced *lac* cells, grown in lactose conditions for 4 h, were exposed to MMM+0.4% glucose for a time 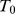, and then switched to a MMM+0.4% lactose environment. The growth rate of the population following the glucose/lactose transition was used to determine the duration of the lag and recovery phases (Fig. S3). The duration of the lag+recovery phases was measured when 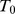 is 4h, 5.5h, 7h, 9h or 12h, or when cells are grown without ever being exposed to lactose (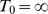). Fig. 2c shows that *lac*-induced cells retained their ability to grow on lactose for 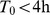, and went through a progressively longer lag phase as 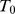 increased.

**Figure 3:**
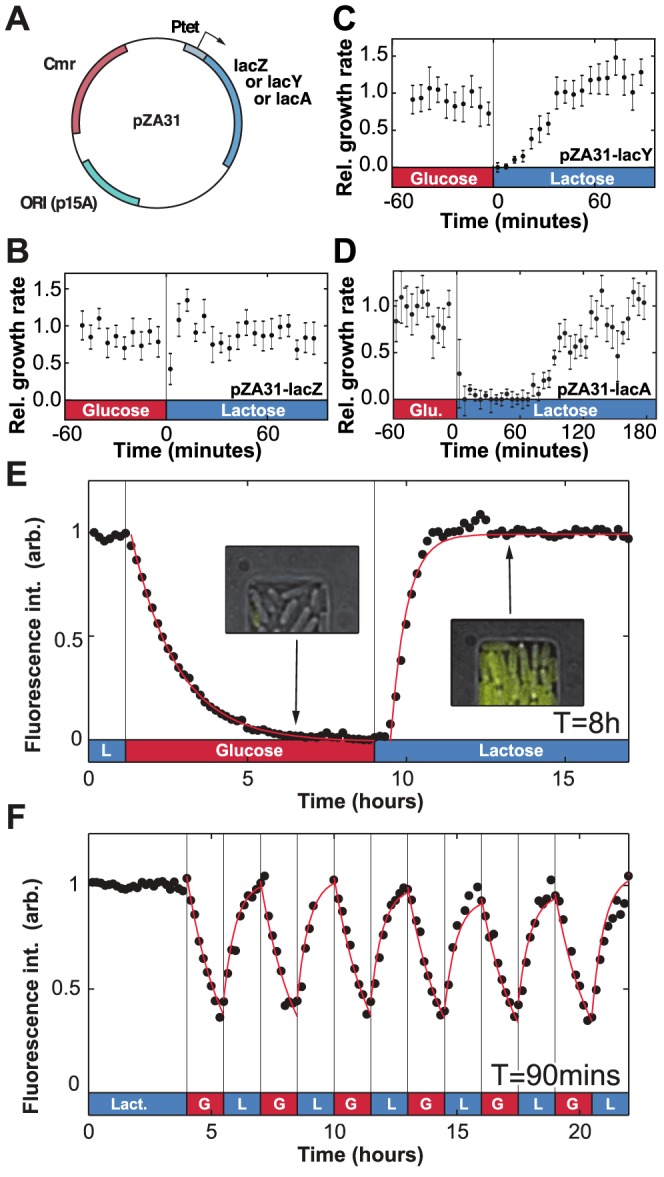
Molecular components of phenotypic memory in the *lac* operon. A) Representation of the over-expression plasmids based on the Lutz and Bujard expression system [43]. B) The lag+recovery phases last less than 10 minutes when cells over-express LacZ. C) LacY over-expression shortens by approximately 15 minutes the duration of the lag+recovery phases following a glucose/lactose transition. D) For LacA over-expression the lag+recovery phases typically last longer than 60 minutes. Data is binned over 5-minute intervals. Error bars: SEM with N = 5 GCs. E) LacY-Venus fusion proteins are used to track the intracellular Lac protein levels. The *in vivo* LacY-Venus levels decrease to zero when glucose and lactose alternate every 8 hours. F) A residual protein level remains within the cells when the environmental duration is 90 minutes. The induction and decay dynamics are accurately described by exponential functions (red lines). Decay in panel E was fit to a form 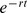, where 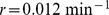; induction was fit to the form 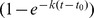 for 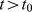, where 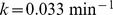 and 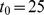 minutes. Red lines in panel F are a plot of the fit found from panel E, starting at each period from the measured initial values.

To identify the proteins that confer phenotypic memory in the *lac* operon (LacZ, LacY or LacA), we measured the duration of the lag+recovery phases for cells that constitutively express one of the three *lac* operon genes. Each gene is driven by the Ptet promoter (Fig. 3a) and details about the over-expression constructs (pZA31-lacZ, pZA31-lacY and pZA31-lacA in Fig. 3b–d) are included in the Materials and Methods section. The duration of the lag+recovery phases for pZA31-lacZ cells, which over-expressed the *β*-galactosidase enzyme, was less than 10 minutes (Fig. 3b), while the lag+recovery phases of pZA31-lacY cells, which over-expressed the lactose permease, lasted approximately 40 minutes (Fig. 3c). Over-expression of *lacA*, the thiogalactoside transacetylase, introduced additional variability in the cellular response between populations and the lag+recovery phases typically lasted longer than 60 minutes (Fig. 3d), ruling out LacA's role in phenotypic memory. These results indicate that high levels of LacZ, and to a lesser extent LacY, are sufficient to maintain cells in an induced state.

Since LacZ and LacY have very low degradation rates (respectively 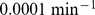 and 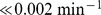 [24], [25]) the main factor that decreases internal levels of *lac* proteins is dilution due to cell growth. For comparison, we note that the *generation time* – the time for the population to double in size – in minimal medium is approximately 60 minutes (see [26], Text S1, and Fig. S2). The maintenance of phenotypic memory should therefore be limited by the number of residual proteins transmitted between the mother and daughter cell during cell division, and phenotypic memory may have an intrinsic lifetime which is tied to the minimal *lac* protein concentration necessary to maintain cells in an induced state.

To confirm this hypothesis, we next measured the *in vivo lac* protein dynamics during lactose/glucose fluctuations using a strain expressing functional LacY-Venus fusion molecules [27]. In Fig. 3e, when lactose and glucose alternated with an environmental duration 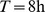, the LacY-Venus permease density decayed to its baseline level with a half-life of 60 minutes, confirming that Lac levels decreased mainly through dilution by growth. Following the reintroduction of lactose, LacY production resumed after a 25 minute lag and reached its half-maximal value in 21 minutes. When 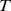 was decreased to 90 minutes, expression of the *lac* operon was modulated by the environmental fluctuations but did not decay to zero (Fig. 3f) and cells maintained a residual intracellular *lac* protein level in the absence of lactose.

In Fig. 2c, the duration of the lag+recovery phases converged toward the 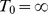 value when 12 hours or more separated lactose exposures. This suggests that the level of *lac* proteins transmitted upon cell divisions was insufficient to maintain cells in a fully or partially induced *lac* state after 10–12 generations. Furthermore, the observation that the same population subjected to many glucose/lactose fluctuations still underwent a lag phase when lactose was removed for more than 4 hours indicates that memory of lactose adaptation does not result from the evolution and fixation of constitutive mutants within the population.

### Probing lag and recovery phases using rapid fluctuations

**Figure 4:**
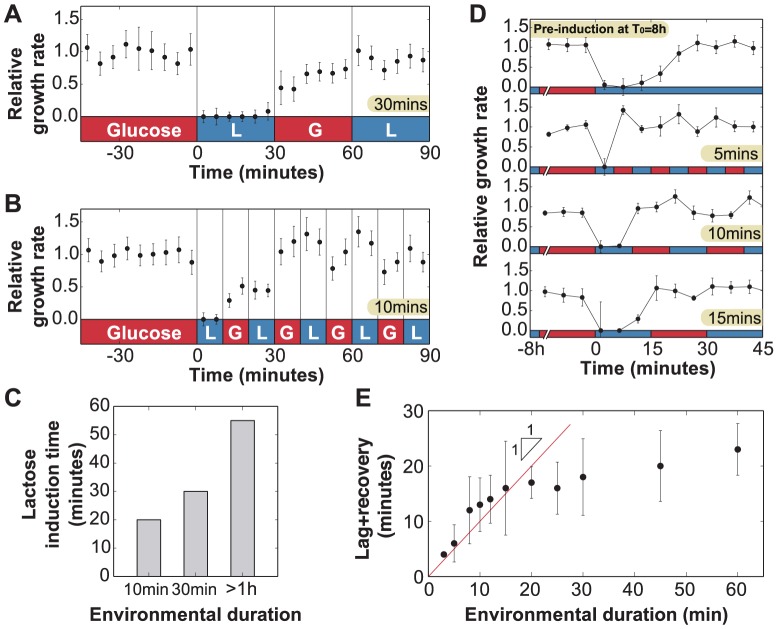
Lag phase and recovery in rapidly fluctuating environments. A) When glucose and lactose alternate with environmental duration *T* = 30 minutes, uninduced cells (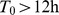) enter a lag phase following the first exposure to lactose only. No lag phase is measured at *t* = 60 minutes, indicating that cells are fully induced when lactose is reintroduced. B) Similarly, when the environmental duration is 10 minutes, cells only spend the first lactose exposure in a lag phase (N = 5 GCs, error bars  =  SEM). C) Amount of time uninduced cells were exposed to lactose conditions before reaching complete growth recovery. Under environmental durations *T* = 10 and 30 minutes, cells recover following 20 and 30 minutes of lactose exposure, respectively, compared with the 55 minutes necessary when the environmental duration 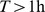. D) Cells pre-induced at 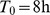 have a 25 minutes lag+recovery phase (N = 5 GCs, error bars  =  SEM). Exposure to rapidly fluctuating glucose/lactose conditions increases adaptation rate, and cells are able to recover after only one exposure to lactose for environmental durations *T* =  5, 10 and 15 minutes. E) Measured lag+recovery times for 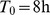 pre-induced cells exposed to fluctuating conditions with an environmental duration *T* = 3-60 minutes. Error bars  =  standard error obtained from lag+recovery regression parameters. All datapoints from *T* = 3–15 minutes indicate that cells recover shortly after glucose is reintroduced (red line indicates cases where the lag+recovery times and *T* are equal), demonstrating that cells are able to fully grow on lactose after a single exposure to lactose conditions.

We analyzed the determinants of lag and recovery phases by exposing uninduced cells to faster fluctuations with an environmental duration *T* = 10 or 30 minutes, which is significantly shorter than the 55 minutes required for full adaptation in constant lactose. For 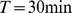 (Fig. 4a), only the first exposure to lactose resulted in cessation of growth; when lactose was reintroduced after 60 minutes, no lag phase was present and cells were fully able to grow under lactose conditions. We observed the same behavior in Fig. 4b for 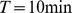, where the lag phase still lasted as long as 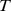 but growth started to recover under glucose conditions and continued to increase through the second exposure to lactose. Cells growing in a rapidly fluctuating environment, *T* = 10 and 30 minutes, were able to grow optimally after only 20 and 30 minutes of lactose exposure respectively, compared to 55 minutes for constant lactose (Fig. 4c).

These experiments yield two key observations. First, the total time to adapt to lactose is shorter when glucose alternates with lactose during the adaptation process. Second, during the glucose exposures (Figs. 4a, 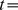
30–60 min; 4b, 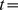
10–20 min), cells are able to resume growth but at a reduced rate. We therefore hypothesized that the lag phase is due to two major barriers that must be crossed before cells can resume normal growth: (A) initiation of *lac* protein production, and (B) recovery from the stringent response caused by carbon starvation [15], [28]. Barrier A consists of de-repression of the *lac* operon, *lac* transcript production, and translation of the first functional LacZ and LacY molecules, which enable subsequent positive feedback. Since these initial events occur in series once the *lac* operon is stochastically de-repressed [27], there exists a certain minimum time to cross the first barrier. In contrast, recovery from the stringent response is only complete once the accumulated (p)ppGpp has decreased to its basal level – barrier B therefore gets longer the more time cells spend without glucose [28].

To test this hypothesis, we attempted to reduce barrier A by starting with a small amount of *lac* protein initially, but not enough to completely eliminate the lag. From Fig. 2c, we know that cells avoid going through a lag phase when grown for up to 4 hours in glucose, i.e. about 4 cell divisions, hence their *lac* proteins are more than 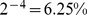 induced; we infer that *lac* levels are sufficiently high to prevent stringent response during lactose exposures once cells have crossed this threshold. We grew *lac*-induced cells under glucose conditions for 8 hours (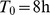), which diluted their *lac* proteins to 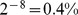 of their maximal level, before beginning rapid lactose-glucose fluctuations. The measured duration of the lag+recovery phase for pre-induced cells was approximately 25 minutes (Fig. 4d, top panel), in agreement with the lag times shown in Fig. 2c, confirming that the lag phase was reduced but not eliminated. When the pre-induced cells were exposed to 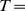 5, 10 or 15 minute fluctuating conditions, they experienced a lag phase only during the first lactose exposure (Fig. 4d), and immediately recovered their ability to fully grow on both glucose and lactose. We plot the lag+recovery times across different fluctuation regimes spanning environmental durations *T* = 3–60 minutes in Fig. 4e. We observed that for rapid fluctuations (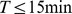) the total adaptation time was approximately equal to the duration of the lactose exposure 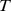 (red line in Fig. 4e). This indicates that we have minimized barrier A (which normally takes a fixed amount of time), and we are mainly seeing barrier B (which is proportional to the duration of carbon stress). For 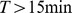, cells are able to resume normal growth in lactose hence barrier B is crossed before the glucose exposure.

Our biological model makes several predictions, which are confirmed in the following section through direct measurements of cytoplasmic *lac* levels. First, Fig. 4d shows that for 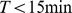 cells do not cross the barrier B threshold (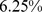 induction) during the initial lactose exposure, but are able to cross it during the glucose exposure; while the cells must be able to maintain their metabolic state using phenotypic memory, the very rapid adaptation suggests that cells may also continue adapting to lactose during the glucose exposures. Second, the initiation of *lac* protein production – barrier A – is a process with a fixed timescale that does not depend on the duration of carbon stress. Third, once barrier A is crossed, barrier B can be crossed in as little as 3 minutes (Fig. 4e, 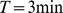). We test these predictions in the next section by using a LacY fluorescent protein fusion to measure the dynamics of *lac* induction and we further elaborate on this simple biological model of the lag phases in the Discussion.

### Response memory and dynamics of *lac* protein expression

**Figure 5:**
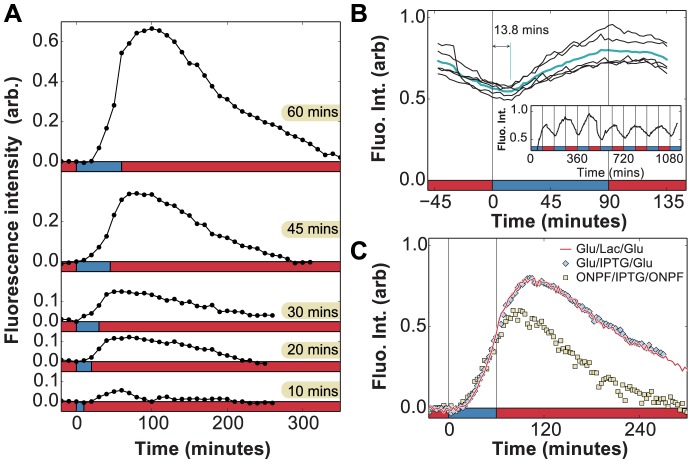
*In vivo* measurement of LacY expression in fluctuating environments. A) Induction dynamics in response to a single pulse of lactose lasting 10, 20, 30, 45, or 60 minutes. In each case, the permease density continues to increase and levels start to decay approximately 40 minutes after lactose is removed from the environment. Experiments were performed with 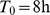 of glucose between induction events (x-axis: red  =  glucose, blue  =  lactose). B) Measurement of phase difference between the glucose/lactose environment and expression level (cyan line  =  average over 5 periods) due to LacY-Venus protein production and maturation times. Measurements from a long experiment in a 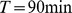 periodic environment (inset) are superimposed onto a single period. The LacY-Venus reporter delay lasts 13.8 minutes on average (x-axis: red  =  glucose, blue  =  lactose). C) Induction by either 60 minutes of 0.4% lactose (red line, x-axis: red  =  glucose, blue  =  lactose) or 1 mM IPTG (blue diamonds, x-axis: red  =  glucose, blue  =  glucose + 1 mM IPTG) leads to similar *lac* induction profiles. The anti-inducer ONPF decreases the duration of response memory, causing *lac* levels to peak only 20 minutes after inducer removal (yellow squares, x-axis: red  =  glucose + 1 mM ONPF, blue  =  glucose + 1 mM IPTG).

To reproduce the conditions of Fig. 4, we measured the changes in LacY-Venus protein levels in response to short (10–60 min) lactose exposures. Subjecting cells to a single pulse of lactose – instead of cyclical fluctuations – ensured that induction dynamics were measured independently of other effects, such as starvation and the stringent response, which may be compounded by multiple glucose-to-lactose transitions. In Fig. 5a, we observed continued production of *lac* proteins after each lactose pulse: LacY-Venus levels continued to rise in the absence of lactose and started to decrease approximately 40 minutes *after* lactose was removed from the environment. These results confirm our conclusion, based on growth measurements in Fig. 4, that *lac* induction continues during the glucose environments following lactose.

We term this behavior *response memory*: the ability of a regulatory network to continue to respond after the stimulus has been removed. Hysteresis and expression delays are to be expected in multi-level gene regulatory circuits, and in the particular case of *lac* regulation these delays can involve the kinetics of mRNA degradation [29], repressor re-binding [30], [31], catabolite repression mediated by cAMP [32], and dynamics of allolactose, the intracellular inducer of the *lac* operon [33]. We therefore characterized the relative contributions of these effects to the observed response memory.

First, the ability to detect *in vivo* changes in *lac* protein levels is set by the maturation time of LacY-Venus (both folding and chromophore formation, measured to last less than 7 minutes *in vivo* [34]), which introduces a delay between observed and actual protein levels. To accurately measure the delay associated with the LacY-Venus protein maturation, we analyzed the LacY-Venus fluorescence levels when glucose and lactose environments alternate with an environmental duration of 90 minutes and measured the phase difference between the environment and the LacY-Venus levels. Since no lag phase is observed for cells under 90 minutes glucose/lactose fluctuations (Fig. 2c), reporter delays do not result from temporary carbon starvation or decreased protein production during a lag phase, and are due solely to the reporter maturation time. The average delay measured under these experimental conditions is 13.8 minutes (Fig. 5b), which is an upper bound on the LacY-Venus maturation time since it includes both maturation time and protein production time. The observed peak at 40 minutes in Fig. 5a is therefore only partially accounted for by reporter delay.

We carried out experiments in which cells grown in MMM+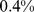 glucose were subjected to a 60 minutes pulse of MMM+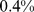 glucose+1 mM of the unmetabolizable inducer IPTG, which yielded identical results to the induction by lactose (Fig. 5c). In contrast to induction using lactose, which requires LacZ activity to produce the inducer allolactose, IPTG induces the *lac* operon directly. Moreover, while cells under glucose/lactose fluctuations experience fluctuating levels of glucose-mediated catabolite repression, constant glucose levels in the IPTG experiments eliminate this effect. Hence, we see that neither lactose/allolactose metabolism nor changes in catabolite repression are required for the observed overshoot. Furthermore, this experiment shows that the stringent response caused by carbon starvation does not significantly affect the induction dynamics.

We next tested whether residual intracellular inducer could account for the observed response memory, by using 2-nitrophenyl *β*-D-fucopyranoside (ONPF), an anti-inducer molecule that competitively binds LacI, excludes IPTG, and increases LacI's affinity for its operator site. In Fig. 5c, cells grown in the presence or absence of 1 mM ONPF exhibited nearly identical induction profiles under IPTG (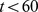 minutes). However, they exhibited significantly different response memory profiles when the inducer was removed at 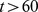 minutes: *lac* expression in the presence of ONPF started to decrease 20 minutes after IPTG removal, compared to the 40 minutes measured in the absence of ONPF. Residual intracellular LacI-bound inducer could therefore account for at least 20 minutes of sustained response in the IPTG/glucose and lactose/glucose experiments. The remaining 6 minutes of response memory, not accounted for by reporter delay, can be explained by the measured time for LacI to fully rebind *lac* operator sites in the presence of ONPF (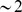 min, [30], [31]) as well as the lifetime of *lac* mRNA (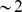 min, [29]).

These *in vivo* measurements support our predictions above based on the growth rate dynamics. First, we found that response memory enables cells to continue responding to lactose through the glucose exposures. Second, we showed in Fig. 5c that the initiation of *lac* protein production is a process with a fixed time that does not depend on the duration of carbon stress. We note that because our experiment is not designed for single-molecule sensitivity, we cannot measure the initiation events themselves. However, we clearly see that cells cross our detection threshold at approximately the same time when induced with IPTG in glucose (i.e. without any carbon stress) or with lactose under carbon stress. Third, we measured the post-initiation rate constant for *lac* protein production to be 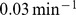. This implies that post-initiation the time to increase *lac* induction levels to 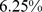 would be approximately 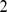 minutes, which is consistent with our prediction that barrier B can be crossed in as little as 3 minutes.

### Modeling *lac* operon dynamics with memory in a fluctuating environment

**Figure 6:**
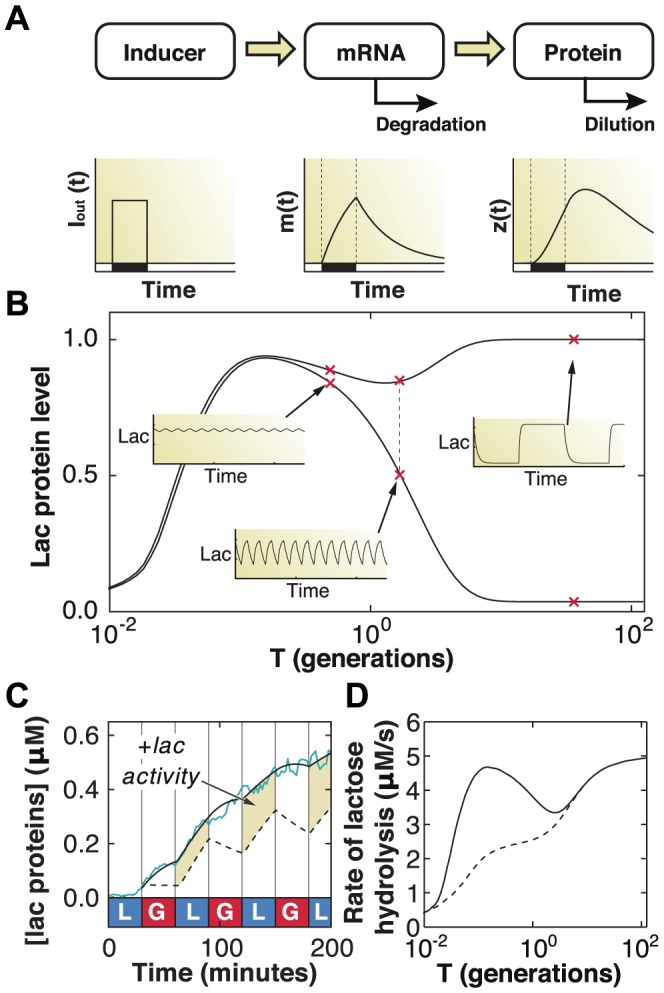
Mathematical modeling quantifies fitness advantage of memory. A) Schematic of the gene regulation model, including extracellular inducer (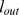), mRNA (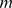), and protein (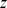). B) The maximum and minimum (top and bottom dashed curves, respectively) *lac* protein concentrations depend on the environmental duration 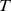, shown in units of generation time (1 generation  =  60 minutes). A few representative examples of how *lac* levels evolve under fluctuating conditions are shown in the insets. C) A difference in *lac* expression levels is observed for models with (solid line) and without (dashed line) response memory. The model that includes response memory correctly predicts the experimentally measured IPTG induction dynamics (cyan line). Response memory leads to increased intracellular LacZ levels and higher catabolic activity. D) The lactose hydrolyzed per unit time at equilibrium during a complete glucose/lactose cycle (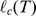) is compared between the two models (dashed line: no response memory; solid line: response memory). Cells making use of response memory consume up to 100% more lactose under rapid fluctuations.

While the major determinants of the lag phase were found to be the initial induction steps and the recovery from stringent response, the potential fitness gains that cells might reap from response memory remained unclear. To better quantify the fitness advantage of response memory in the *lac* operon, we adapted the established metabolic model described in [35] to fluctuating environments (see Materials and Methods). We focused exclusively on the observed memory effects and their impact on metabolic activity, and did not model the stringent response since it did not significantly affect the induction dynamics (Fig. 5c). The model explicitly accounts for intracellular concentrations of lactose, allolactose, *lac* operon mRNA, and *lac* proteins, and captures several features we observed in experiments. For example, in response to a single pulse of extracellular inducer, protein levels can continue to increase after the stimulus is removed, causing an overshoot, if sufficient mRNA and intracellular inducer levels are maintained (Fig. 6a). Likewise, the model exhibits phenotypic memory consistent with our observations. Fig. 6b shows the minimum and maximum *lac* protein levels at equilibrium as a function of the environmental duration 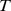 in units of generation time (measured in the bulk in [26] to be 60 minutes in MMM+glucose).

Since response memory can be explained by the LacI-mediated repression kinetics (Fig. 5c), and given that the timescales for LacI rebinding to the operator have been measured to be only a few minutes [30], [31], our data suggest that there is sufficient residual allolactose inside the cell for sustained expression. We used the model to test this conclusion by artificially reducing the allolactose degradation rate to zero during glucose environments. We obtained similar results across a range of slower but non-zero degradation rates. We show in Fig. 6c the *lac* protein levels predicted by solving the model with (solid line) and without (dotted line) residual inducer, the latter yielding the response memory behavior in which cells continue adapting through the glucose exposures. The predicted dynamics closely follow the measured *lac* levels obtained from 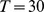 minutes IPTG induction (cyan line). The highlighted regions in Fig. 6c correspond to excess metabolic activity, which we compute by integrating the total amount of lactose consumed over a full glucose/lactose cycle at equilibrium (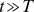). We find that cells with response memory exhibit an increased capability to metabolize lactose following short exposures to lactose and, if the fluctuating conditions were to persist, are able to hydrolyze up to 100% more lactose when the environment fluctuates faster than the typical generation time (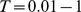 generations, Fig. 6d). The modeling results support a picture in which response memory provides a large adaptive advantage when external fluctuations occur faster than the cell division time, while phenotypic memory is beneficial for slower fluctuations, spanning several generations.

## Discussion

We have presented two distinct memory mechanisms in the *lac* operon of *E. coli*, phenotypic and response memory, each of which is beneficial over different timescales. Phenotypic memory allows cells to maintain an adapted state for multiple generations after a specific carbon source is removed from the environment. Since phenotypic memory operates through the transmission of stable cytoplasmic proteins, it may be employed as a general strategy in other organisms to transmit metabolic information between generations, as observed e.g. in the yeast galactose system [9], [36]. More generally, the intrinsic mechanism behind phenotypic memory being passive – based on intracellular proteins whose lifetime is longer than a typical generation – similar memory effects are expected to be present for other fluctuations and in other organisms. Adaptation mechanisms that rely on the expression of long-lived permease molecules – e.g. small molecule transport [37] or antibiotic/toxin efflux systems [38] – or the production of enzymatic components whose activity confers a distinct fitness advantage such as sigma factors involved in stress response systems [39] constitute examples of phenotypic memory mechanisms.

We used fast fluctuating environments to dissect the determinants of lag phases following a transition from glucose to lactose. Our results suggest a simple biological model of the lag phase in which *lac* protein activity and the stringent response are mutually inhibitory processes: *Lac* protein activity in lactose has an inhibitory effect on the stringent response due to glucose production and amino acid synthesis, while the stringent response initially inhibits *lac* protein production through its global inhibitory effects on translation. To see this, we consider two examples. First, we compare 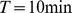 for uninduced and pre-induced cells (Fig. 4b+d). In the pre-induced case, after the first lactose exposure cells rapidly recover full growth in glucose, whereas if no *lac* proteins are initially available, cells experience a slow recovery in glucose. The stringent response due to the lactose exposure is therefore much less severe when a small amount of LacZ (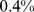 induced) is available to hydrolyze lactose and initiate positive autoregulation. Second, we note that for 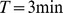 (Fig. 4e), full adaptation to lactose is achieved by the second lactose exposure, which means that *lac* protein levels can cross the 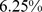 threshold within 6 minutes total. However, for 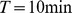 (Fig. 4d), the cells do not begin growing during the first lactose exposure even though more than 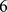 minutes have elapsed. We conclude that protein production during the stringent response is too slow to allow cells to cross the threshold during the short lactose exposures for 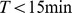.

While cells under fast glucose/lactose fluctuations could in principle become constitutively active and never repress the *lac* operon, the metabolic cost associated with unnecessary *lac* expression would incur a significant fitness disadvantage [40]. In particular, if lactose encounters unexpectedly cease, this cost will no longer be temporary, but sustained by the population indefinitely. Cells employing response memory avoid such long-term cost by *transiently* expressing the required genes for a short amount of time following an initial exposure to the stimulus, with a maximal metabolic cost that is limited by the duration of this transient expression. Should environmental fluctuations cease, cells will suffer only a small, short-term fitness cost. On the other hand, should fast fluctuations persist, as we have shown the cells reap a significant fitness benefit. In particular, we showed that cells reach higher induction levels more rapidly by maintaining their response profile following the removal of an external inducer.

Memory in different genetic network architectures could affect not only the cost of gene expression, but also the evolution of gene expression levels. The timescale over which phenotypic memory persists is determined to a large extent by the gene's expression level (provided the protein is sufficiently stable). Expression levels may be evolutionarily tuned not only to support growth in a single environment, but also to provide cells' progeny with memory of past environments. The interplay of memory and metabolic constraints could thus dramatically change the nature of evolutionary trajectories and optima. We expect theoretical analyses may be fruitfully applied to explore these possibilities.

The power of the memory mechanisms we have described lies in their universality. Protein lifetimes and regulatory networks can be tuned in simple ways to give rise to physiological memory under rapidly changing conditions. Microorganisms have to handle both internal and external sources of noise, and while many genetic networks have evolved to exploit stochastic fluctuations of intracellular molecular components to regulate key cellular processes [41], we have shown that molecular rates of signal transduction reactions can be modulated to optimize response profiles for growth in fluctuating environments. Together, phenotypic and response memory allow bacteria to adapt to a wide range of fluctuation timescales in sophisticated, history-dependent ways. These memory mechanisms constitute general strategies that bacteria can employ to adapt to diverse environmental fluctuations – including nutrients, antibiotics, and other physiological stresses.

## Materials and Methods

### Device description and fabrication

The microfluidic device used in this study was made using standard soft lithography and microfabrication techniques and consists of growth chambers and a main flow channel patterned from two SU-8 layers 1.1 microns (SU-8 2, spun at 3000 rpm) and 20 microns (SU-8 2025, spun at 4000 rpm) in height, respectively. The devices were fabricated by making polydimethylsiloxane (PDMS) replicates of the SU-8 master. The PDMS devices were peeled from the silicon master and 1.5 mm holes were punched (Harris Uni-core, Ted Pella) to create the input and output ports and each individual device was bonded to a glass bottom petri dish (PELCO Glass Bottom Dishes, Ted Pella) using an oxygen plasma treatment. 16ga needles attached to tygon tubing (TYGON tubing, Cole Parmer) were inserted into each port and inline solenoid valves (two-way normally closed 1/16 12VDC, Cole Parmer) were used to control liquid flow inside the device from pressurized reservoirs. A flow rate of 5 mL/h, which corresponds to a flow speed of 30 cm/s inside the main channel, was used by applying a 6psi pressure to the reservoirs. When transitioning between two media, both valves were closed for 15 seconds before the new one was opened to let the pressure equilibrate inside the device and to avoid backflow problems. A T-junction upstream of the growth chambers ensured that transitions between the different media occurred very rapidly. By flowing a fluorescent dye inside the device, the transition between each type of media was measured to occur in less than 250 milliseconds (Supp. Video S1) in the main flow channel and no residual flow from the “off” inlet port was observed.

### Growth rate measurements

Cells inside the growth chambers push their immediate neighbors toward the main flow channel as they increase in size, and the lateral speed 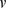 at which the cells move is proportional to the population's mean elongation rate. An optical flow algorithm implemented using openCV [42] was used to measure the displacement between successive frames. This displacement was used to find the average cell speed 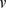 over the region between 10 and 15 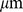 away from the closed end of the growth chamber. The cell speed was then averaged over a 5 minute time-window, averaged over the 5 chambers present in the image, and scaled relative to the speed measured under MMM+0.4% glucose conditions. The lateral speed reports on the cumulative growth rate of cells in the first 10 microns of the growth chamber providing a measure of the relative growth rate of the population. Error bars on relative growth plots report the standard error of the mean as averaged over the 5 chambers present in a single field of view. These error bars measure intrinsic cell-to-cell variability in growth, due to stochasticity in cell division rates, elongation rates, and gene expression processes.

### Mathematical model of lactose metabolism

The *lac* induction dynamics of a population subjected to sudden environmental changes are modeled as described in [35], with an additional equation to account for mRNA transcription. The model assumes that LacY protein levels are proportional to LacZ levels. Unless otherwise noted in Table 1, refer to [35] for a complete rationalization behind each parameter's value. The set of equations are given by
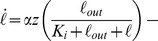


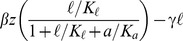
(1)

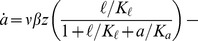


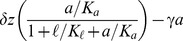
(2)

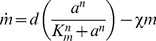
(3)

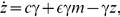
(4)where 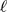, 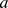, 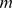, and 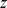 are the intracellular concentrations of lactose, allolactose, mRNA and LacZ proteins, respectively (parameters are specified in Table 1).

**Table 1:** Model parameters.

Symbol	Description	Value
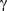	growth rate	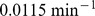
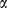	permease import turnover number	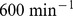
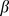	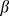 -gal lactose turnover number	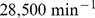
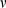	lactose 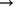 allolactose branching ratio	0.448
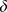	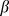 -gal allolactose turnover number	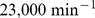
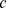	basal 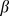 -gal level	34.2 nM
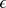	fully induced 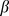 -gal level	34,286 nM
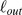	external lactose cencentration	11,700 nM
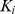	permease Michaelis constant	5×10 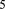 nM
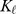	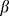 -gal lactose Michaelis constant	2,530 nM
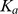	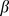 -gal allolactose Michaelis constant	1,200 nM
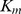	half-maximal *lac* induction level	10 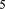 nM
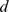	mRNA transcription rate	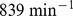
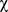	mRNA degradation rate	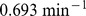
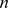	Hill number for *lac* induction	2

Parameters used in the mathematical model of the *lac* operon. Unless otherwise noted, refer to [35] for a description of the parameter values. The growth rate 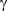 corresponds to a generation time of 60 minutes (i.e. without the fitness costs associated with GFP and KanR production).

Model equations were solved numerically for cyclical glucose/lactose conditions with an environmental duration 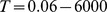 minutes. After a time 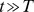, the *lac* expression levels immediately before a glucose/lactose (lactose/glucose) change are recorded to obtain the minimum (maximum) *lac* protein level. The expression for the lactose hydrolysis rate 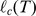 is given by
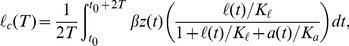
(5)where 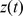, 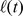, and 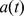 are obtained by solving Eqns. 1-4, and 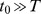.

To qualitatively compare behaviors with and without response memory, we artificially reduced the rate of allolactose turnover in glucose environments (taking 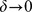) to attain response memory in our simple model. Similar results were obtained by reducing instead the mRNA degradation rate in the transition from lactose to glucose.

**Full methods** as well as further details of microfluidic fabrication, strain description, image acquisition and analysis, and any associated references are provided in Text S1.

## Supporting Information

Figure S1Timescale of the environmental change inside a chemoflux. Fluorescence levels measured in the growth chamber following DI water/DI water + fluorescein media transitions. The transition are accurately described by exponential functions (red lines, 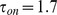 seconds and 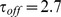 seconds).(PDF)

Figure S2Growth rate measurement. The growth rate of the population is extracted from the cell-cycle age distribution of cells growing inside GCs under constant MMM+0.4%glucose conditions. Since each cell division event yields two cells at age zero, the fraction of cells at age 0 is twice the population's growth rate. The age of cells growing in 5 GCs over 200 minutes is combined to find, from the fraction of cells at age 0, a population growth rate 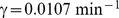 (generation time  =  64.7 minutes).(PDF)

Figure S3 Duration of the lag phase. A) The duration of the lag+recovery phase is monitored for cells that encounter lactose for the first time in more than 24 hours. Cells with a fully induced *lac* operon are exposed to MMM+0.4% glucose for 12h, 9h, 7h, 5.5 h and 4 h. B) - F) The duration of the lag and recovery phases is computed from a linear regression of the lateral cell speed and the results are presented in Fig. 2c.
(PDF)

Text S1 Supplementary methods.
(PDF)

Video S1 A video showing transition of media inside the chemoflux from DI water to DI water + fluorescein (see Fig. S1 for quantification).
(MOV)

Video S2 A video of cell growth in the chemoflux growth chambers during a glucose-to-lactose transition.
(MOV)

## References

[1] Monod J (1942) Recherches sur la croissance des cultures bactériennes. Paris: Hermann & cie.

[2] Monod J (1949) The growth of bacterial cultures. Annu Rev Microbiol 3: 371–394.

[3] Dekel E, Alon U (2005) Optimality and evolutionary tuning of the expression level of a protein. Nature 436: 588–592. 16049495 10.1038/nature03842

[4] Magasanik B (1961) Catabolite repression. Cold Spring Harb Sym 26: 249–256. 10.1101/sqb.1961.026.01.031 14468226

[5] Gorke B, Stulke J (2008) Carbon catabolite repression in bacteria: many ways to make the most out of nutrients. Nat Rev Microbiol 6: 613–624. 18628769 10.1038/nrmicro1932

[6] New A, Cerulus B, Govers S, Perez-Samper G, Zhu B, et al (2014) Different levels of catabolite repression optimize growth in stable and variable environments. PLoS Biol 12: e1001764. 24453942 10.1371/journal.pbio.1001764 PMC3891604

[7] Scott M, Gunderson CW, Mateescu EM, Zhang Z, Hwa T (2010) Interdependence of cell growth and gene expression: Origins and consequences. Science 330: 1099–1102. 21097934 10.1126/science.1192588

[8] Deris JB, Kim M, Zhang Z, Okano H, Hermsen R, et al (2013) The innate growth bistability and fitness landscapes of antibiotic-resistant bacteria. Science 342: 1237435. 24288338 10.1126/science.1237435 PMC4059556

[9] Razinkov IA, Baumgartner BL, Bennett MR, Tsimring LS, Hasty J (2013) Measuring competitive fitness in dynamic environments. J Phys Chem B 117: 13175–13181. 23841812 10.1021/jp403162v PMC3808460

[10] Boulineau S, Tostevin F, Kiviet DJ, ten Wolde PR, Nghe P, et al (2013) Single-cell dynamics reveals sustained growth during diauxic shifts. PLoS ONE 8: e61686. 23637881 10.1371/journal.pone.0061686 PMC3640066

[11] Casadesús J, D'Ari R (2002) Memory in bacteria and phage. BioEssays 24: 512–518. 12111734 10.1002/bies.10102

[12] Norman TM, Lord ND, Paulsson J, Losick R (2013) Memory and modularity in cell-fate decision making. Nature 503: 481–486. 24256735 10.1038/nature12804 PMC4019345

[13] Wolf DM, Fontaine-Bodin L, Bischofs I, Price G, Keasling J, et al (2008) Memory in microbes: Quantifying history-dependent behavior in a bacterium. PLoS ONE 3: e1700. 18324309 10.1371/journal.pone.0001700 PMC2264733

[14] Alon U (2007) Network motifs: theory and experimental approaches. Nat Rev Genet 8: 450–461. 17510665 10.1038/nrg2102

[15] Chang DE, Smalley DJ, Conway T (2002) Gene expression profiling of Escherichia coli growth transitions: an expanded stringent response model. Mol Microbiol 45: 289–306. 12123445 10.1046/j.1365-2958.2002.03001.x

[16] Magnusson LU, Farewell A, Nyström T (2005) ppGpp: a global regulator in Escherichia coli. Trends Microbiol 13: 236–242. 15866041 10.1016/j.tim.2005.03.008

[17] Potrykus K, Cashel M (2008) (p)ppGpp: still magical? Annu Rev Microbiol 62: 35–51. 18454629 10.1146/annurev.micro.62.081307.162903

[18] Ferullo DJ, Lovett ST (2008) The stringent response and cell cycle arrest in Escherichia coli. PLoS Genet 4: e1000300. 19079575 10.1371/journal.pgen.1000300 PMC2586660

[19] Barker MM, Gaal T, Josaitis CA, Gourse RL (2001) Mechanism of regulation of transcription initiation by ppGpp. i. effects of ppGpp on transcription initiation in vivo and in vitro. J Mol Biol 305: 673–688. 11162084 10.1006/jmbi.2000.4327

[20] Jablonka E, Oborny B, Molnar I, Kisdi E, Hofbauer J, et al (1995) The adaptive advantage of phenotypic memory in changing environments. Philos T Roy Soc B 350: 133–141. 10.1098/rstb.1995.0147 8577857

[21] Wang P, Robert L, Pelletier J, Dang WL, Taddei F, et al (2010) Robust growth of Escherichia coli. Curr Biol 20: 1099–1103. 20537537 10.1016/j.cub.2010.04.045 PMC2902570

[22] Aldridge BB, Fernandez-Suarez M, Heller D, Ambravaneswaran V, Irimia D, et al (2012) Asymmetry and aging of mycobacterial cells lead to variable growth and antibiotic susceptibility. Science 335: 100–104. 22174129 10.1126/science.1216166 PMC3397429

[23] Loomis WF, Magasanik B (1967) Glucose-lactose diauxie in Escherichia coli. J Bacteriol 93: 1397–1401. 5340309 10.1128/jb.93.4.1397-1401.1967 PMC276614

[24] Mandelstam J (1958) Turnover of protein in growing and non-growing populations of escherichia coli. Biochem J 69: 110–119. 13535591 10.1042/bj0690110 PMC1196522

[25] McKenna E, Hardy D, Pastore JC, Kaback HR (1991) Sequential truncation of the lactose permease over a three-amino acid sequence near the carboxyl terminus leads to progressive loss of activity and stability. P Natl Acad Sci USA 88: 2969–2973. 10.1073/pnas.88.8.2969 PMC51365 2014218

[26] Neidhardt FC, Bloch PL, Smith DF (1974) Culture medium for enterobacteria. J Bacteriol 119: 736–747. 4604283 10.1128/jb.119.3.736-747.1974 PMC245675

[27] Choi PJ, Cai L, Frieda K, Xie XS (2008) A stochastic single-molecule event triggers phenotype switching of a bacterial cell. Science 322: 442–446. 18927393 10.1126/science.1161427 PMC2819113

[28] Murray K, Bremer H (1996) Control of SpoT-dependent ppGpp synthesis and degradation in Escherichia coli. J Mol Biol 259: 41–57. 8648647 10.1006/jmbi.1996.0300

[29] Kennell D, Riezman H (1977) Transcription and translation initiation frequencies of the Escherichia coli lac operon. J Mol Biol 114: 1–21. 409848 10.1016/0022-2836(77)90279-0

[30] Elf J, Li GW, Xie XS (2007) Probing transcription factor dynamics at the single-molecule level in a living cell. Science 316: 1191–1194. 17525339 10.1126/science.1141967 PMC2853898

[31] Hammar P, Leroy P, Mahmutovic A, Marklund EG, Berg OG, et al (2012) The lac repressor displays facilitated diffusion in living cells. Science 336: 1595–1598. 22723426 10.1126/science.1221648

[32] Epstein W, Rothman-Denes LB, Hesse J (1975) Adenosine 3′:5′-cyclic monophosphate as mediator of catabolite repression in Escherichia coli. P Natl Acad Sci USA 72: 2300–2304. 10.1073/pnas.72.6.2300 PMC432745 166384

[33] Huber RE, Kurz G, Wallenfels K (1976) A quantitation of the factors which affect the hydrolase and transgalactosylase activities of *β*-galactosidase (e. coli) on lactose. Biochemistry-US 15: 1994–2001. 10.1021/bi00654a029 5122

[34] Nagai T, Ibata K, Park ES, Kubota M, Mikoshiba K, et al (2002) A variant of yellow fluorescent protein with fast and efficient maturation for cell-biological applications. Nat Biotechnol 20: 87–90. 11753368 10.1038/nbt0102-87

[35] Dreisigmeyer DW, Stajic J, Nemenman I, Hlavacek WS, Wall ME (2008) Determinants of bistability in induction of the Escherichia coli lac operon. IET Syst Biol 2: 293–303. 19045824 10.1049/iet-syb:20080095

[36] Zacharioudakis I, Gligoris T, Tzamarias D (2007) A yeast catabolic enzyme controls transcriptional memory. Curr Biol 17: 2041–2046. 17997309 10.1016/j.cub.2007.10.044

[37] Pao SS, Paulsen IT, Saier MH (1998) Major facilitator superfamily. Microbiol Mol Biol R 62: 1–34. 10.1128/mmbr.62.1.1-34.1998 PMC98904 9529885

[38] Nikaido H (2009) Multidrug resistance in bacteria. Annu Rev Biochem 78: 119–146. 19231985 10.1146/annurev.biochem.78.082907.145923 PMC2839888

[39] Weber H, Polen T, Heuveling J, Wendisch VF, Hengge R (2005) Genome-wide analysis of the general stress response network in Escherichia coli: σ^S^-dependent genes, promoters, and sigma factor selectivity. J Bacteriol 187: 1591–1603. 15716429 10.1128/JB.187.5.1591-1603.2005 PMC1063999

[40] Eames M, Kortemme T (2012) Cost-benefit tradeoffs in engineered lac operons. Science 336: 911–915. 22605776 10.1126/science.1219083

[41] Eldar A, Elowitz MB (2010) Functional roles for noise in genetic circuits. Nature 467: 167–173. 20829787 10.1038/nature09326 PMC4100692

[42] Farnebäck G (2003) Two-frame motion estimation based on polynomial expansion. In: Bigun J, Gustavsson T, editors, Image Analysis, Springer Berlin Heidelberg, number 2749 in Lecture Notes in Computer Science. pp. 363–370.

[43] Lutz R, Bujard H (1997) Independent and tight regulation of transcriptional units in Escherichia coli via the LacR/O, the TetR/O and AraC/I1-I2 regulatory elements. Nucleic Acids Res 25: 1203–1210. 9092630 10.1093/nar/25.6.1203 PMC146584

