# Correction: Memory and Fitness Optimization of Bacteria under Fluctuating Environments

**DOI:** 10.1371/journal.pgen.1004793

**Published:** 2014-10-16

**Authors:** The PLOS Genetics Staff

## Notice of Republication

This article was republished on September 30, 2014, to correct errors in the authorship list and citation that were introduced during the typesetting process. The second author’s name was spelled incorrectly. Please download this article again to view the correct version. The originally published, uncorrected article and the republished, corrected article are provided here for reference.

## Supporting Information

File S1 Originally published, uncorrected article.
(PDF)

File S2 Republished corrected article.
(PDF)
