# Supplementary figures and images for: Memory and Fitness Optimization of Bacteria under Fluctuating Environments
Source: PLoS Genet. 2014 Sep 25;10(9):e1004556. doi: 10.1371/journal.pgen.1004556 (PMC4177670; doi:10.1371/journal.pgen.1004556)

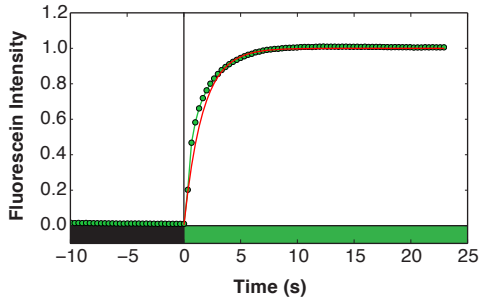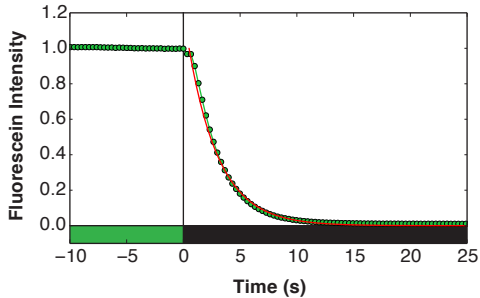

Supplement: Figure S1 — Timescale of the environmental change inside a chemoflux. Fluorescence levels measured in the growth chamber following DI water/DI water + fluorescein media transitions. The transition are accurately described by exponential functions (red lines, seconds and seconds). (PDF) [file pgen.1004556.s001.pdf]

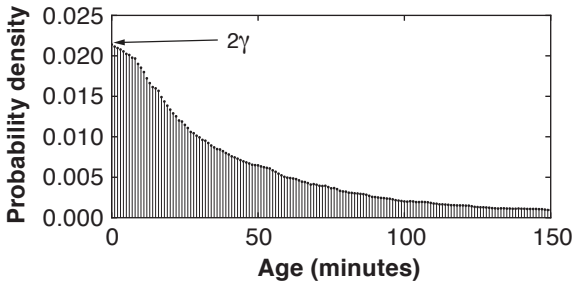

Supplement: Figure S2 — Growth rate measurement. The growth rate of the population is extracted from the cell-cycle age distribution of cells growing inside GCs under constant MMM+0.4%glucose conditions. Since each cell division event yields two cells at age zero, the fraction of cells at age 0 is twice the population's growth rate. The age of cells growing in 5 GCs over 200 minutes is combined to find, from the fraction of cells at age 0, a population growth rate (generation time = 64.7 minutes). (PDF) [file pgen.1004556.s002.pdf]

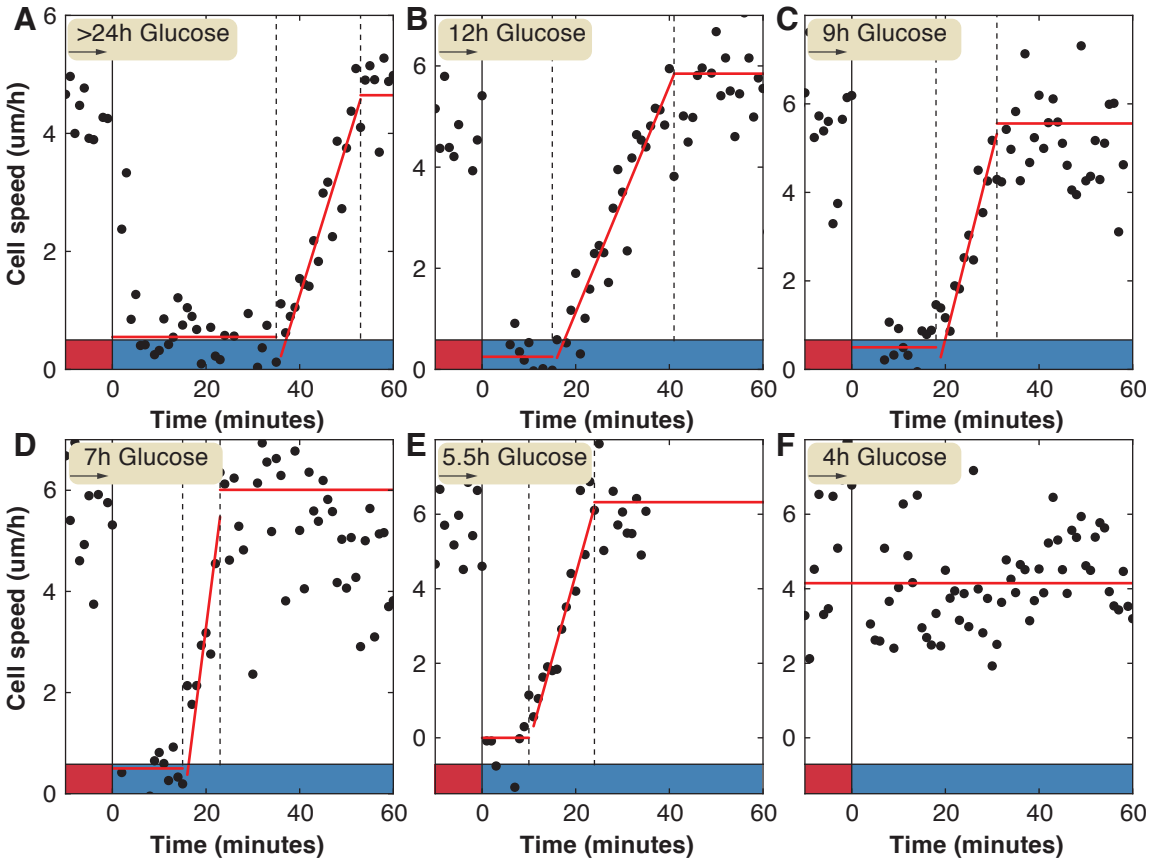

Supplement: Figure S3 — Duration of the lag phase. A) The duration of the lag+recovery phase is monitored for cells that encounter lactose for the first time in more than 24 hours. Cells with a fully induced lac operon are exposed to MMM+0.4% glucose for 12h, 9h, 7h, 5.5 h and 4 h. B) - F) The duration of the lag and recovery phases is computed from a linear regression of the lateral cell speed and the results are presented in Fig. 2c. (PDF) [file pgen.1004556.s003.pdf]
