# Supplementary material for: Memory and Fitness Optimization of Bacteria under Fluctuating Environments
Source: PLoS Genet. 2014 Sep 25;10(9):e1004556. doi: 10.1371/journal.pgen.1004556 (PMC4177670; doi:10.1371/journal.pgen.1004556)
Supplement: Text S1 — Supplementary methods. (PDF) [file pgen.1004556.s004.pdf]

Supplementary Information: Memory and fitness optimization of bacteria under fluctuating environments

Guillaume Lambert[1], Edo Kussell[2,3]

1. The Institute of Genomics and Systems Biology,

The University of Chicago, Chicago, Illinois 60637, USA.

2. Department of Biology and Center for Genomics and Systems Biology,

New York University, New York, New York 10003, USA.

3. Department of Physics, New York University, New York, New York 10003, USA.

SUPPLEMENTARY METHODS

Strain construction and cell culture

Experiments were performed using the non-motile F3 strain of *E. coli*, obtained from the Wakamoto lab (University of Tokyo), which is based on the W3110 background with three genes knocked out – $fliC$, $fimA$, and flu – to decrease cell aggregation and adhesion to surfaces [43, 44]. The strain contained a plasmid expressing GFPmut2 under the control of the $rpsL$ promoter [45] and conferring kanamycin resistance. The three plasmids (pZA31-lacZ, pZA31-lacY and pZA31-lacA) were cloned using KpnI/HindIII and all three genes were amplified from MG1655 using a high-efficiency proofreading polymerase (Phusion, NEB). The plasmids carry a chloramphenicol resistance gene and use a constitutive expression system based on the Lutz and Bujard Ptet expression system [46] with $\sim$ 10 plasmids/cell. A kamanycin resistant strain expresses a functional LacY-Venus fusion protein and has an otherwise intact lac operon (see [47] for details concerning the strain construction). The Venus fluorophore was chosen because of its very fast maturation time [48].

Individual colonies were selected and grown overnight in MOPS minimal media. Cells from the overnight culture containing the necessary selection agent (50 μg/mL of kanamycin or 10μg/mL of chloramphenicol) were diluted by a factor of 100 in MOPS Minimal Media (Teknova) supplemented with 0.4% glucose and grown to an optical density OD$\sim$ 0.5. 4mL of the cell culture was collected by centriguation and approximately 5μL of the cell solution was then injected into the main flow channel through the outlet port. Inlet and outlet tubes were then attached to the device and the device was flushed with fresh media in order to remove the cells from the main flow channel. Cells were grown under constant environmental conditions for 12h before being subjected to the first environmental fluctuation.

Time-lapse microscopy

The Micromanager suite [49] was used to control the microscope stage (H117 ProScan, Prior scientific), the state of the solenoid valves and the camera (Retiga EXi, QImaging). An auto-focus unit (PerfectFocus, Nikon) maintained a constant focus position over the duration of the experiment. An acrylic enclosure surrounding the stage and a heating unit maintained the temperature constant at 37°C throughout the experiment. A high-power LED (Mounted High Power LED,

nominal wavelength 6500K, Thorlabs) was used as the excitation light and fluorescence image were recorded under high magnification (Plan Apo VC 100X, NA=1.4, WD=0.13mm, Nikon) using a CCD camera (RetigaEXi, qImaging). A fluorescence micrograph was recorded every 30 seconds for the lag and fluctuation experiments (2s. exposure time), every 60 seconds for strains over-expressing $lacZ/Y/A$ (2s. exposure time). We used an image stabilization algorithm [50] to correct the frame-to-frame positional drift.

Media transitions

During a typical media transition, in-line solenoid valves are first closed for 15s before the new media is allowed to flow inside the device. This 15s delay ensures that the pressurized media is dissipated from the device and limits residual and back flow problems. The time required to fully transition between two environmental conditions is carefully measured using fluorescein as a tracker molecule. In particular, the fluorescence levels inside the last 10 microns of the growth chambers is monitored as the flow inside the device is switched between distilled water + 0.02% fluorescein and distilled water only.

The fluid flow inside the main channel is laminar and is rapidly replaced by the new media, as shown in supp. video S1. No laminar flow is occurring inside the growth chambers and media transitions are instead dominated by diffusion. The transition time is thus limited by the time required for the fluorescein molecules to diffuse into the growth chambers from the main channel. The measured fluorescence levels at the end of the growth chamber as the media is cycled between DI water and DI water + 0.02 fluorescein is shown in Fig. S**??**. Once the DI water is allowed to flow inside the main channel, the fluorescein levels present inside the growth chamber decays with a time constant $\tau_{off} = 2.7$ seconds. The transition between DI water only and DI water + 0.2% fluorescein exponentially converges with a time constant $\tau_{on} = 1.7$ seconds. The small discrepancy between the τ_{on}/τ_{off} constants are due to the geometry of the device, where each chamber is located along the wall of the main flow chamber and is thus closer to one of the two "branch" of the T-junction.

Lag time measurements

To precisely determine the duration of the lag/recovery phases shown in Fig. 2c and Fig. 4e, the approximate location of the transition between the lag/recovery phase transition and between the recovery phase and full growth were first identified by hand. Then, a linear regression was applied to the data belonging to the manually determined recovery phase, excluding the first and last three points. The lag/recovery and recovery/growth transitions were identified as the time at which the regression equals the growth rate during the lag phase (usually close to zero) and the average speed for the first 20 minutes of the growth phase, respectively (Fig. S3). The uncertainties in the duration of the lag and recovery phases were computed from the standard error of the regression parameters. Fig. S3 shows the resulting linear regressions obtained for each experiment reported in Fig. 2c.

LacY-Venus fluorescence levels

A strain expressing a functional LacY-Venus fusion protein was used to monitor the expression levels of the *lac* operon. To avoid photobleaching of the Venus fluorophore, an image was recorded every 10 minutes for Fig. 3e,f and Fig. 5a, and every 3 minutes for Figs. 5b,c. Cell size did not change during the glucose/lactose transitions and we used the fluorescence intensity recorded over the entire chamber as a measure of the population's expression level. The fluorescence intensity of the 5 chambers within each field of view was averaged and before being plotted. The deviation between the chambers was less than 3% on average.

Growth rate measurements

The growth rate γ of cell populations shown in Fig. S2 was measured inside the growth chambers using cell tracking software [51] to process fluorescence micrographs and identify single cells with each chamber. Each cells was tracked over time and division events were identified. At each time frame, the time since each cell's last division was recorded (i.e. the cell-cycle age) and the age data of 5 growth chambers over 200 minutes under constant MMM+0.4% glucose was combined to generate an age distribution histogram (Fig. S2). The fraction of cells at age 0 was used to measure of the growth rate [44]. Cells growing in the device were found to have a growth rate $\gamma = 0.0107$ min^{-1}. This corresponds to a generation time of 64.8 minutes, slightly longer than the

generation time in bulk measurements (60 minutes, [52]). The slightly lower growth rate inside the GCs may be a consequence of the fitness costs associated in GFPmut2 production, synthesis of KanR proteins, and replication of the pUA66 plasmid in each cell. Since cells do not have to produce these extraneous proteins, the bulk growth rate γ=0.0115 mins (generation time = 60 minutes) was used in the model.

44. Merrin J, Leibler S, Chuang JS (2007) Printing multistrain bacterial patterns with a piezoelectric inkjet printer. PLoS ONE 2: e663.

45. Wakamoto Y, Grosberg AY, Kussell E (2012) Optimal Lineage Principle for Age-Structured Populations. Evolution 66: 115–134.

46. Zaslaver A, Bren A, Ronen M, Itzkovitz S, Kikoin I, et al. (2006) A comprehensive library of fluorescent transcriptional reporters for escherichia coli. Nature methods 3: 623–628.

47. Lutz R, Bujard H (1997) Independent and tight regulation of transcriptional units in escherichia coli via the LacR/O, the TetR/O and AraC/I1-I2 regulatory elements. Nucleic Acids Research 25: 1203–1210.

48. Choi PJ, Cai L, Frieda K, Xie XS (2008) A stochastic single-molecule event triggers phenotype switching of a bacterial cell. Science (New York, NY) 322: 442–446.

49. Nagai T, Ibata K, Park ES, Kubota M, Mikoshiba K, et al. (2002) A variant of yellow fluorescent protein with fast and efficient maturation for cell-biological applications. Nature Biotechnology 20: 87–90.

50. Edelstein A, Amodaj N, Hoover K, Vale R, Stuurman N (2010) Computer control of microscopes using uManager. In: Ausubel FM, Brent R, Kingston RE, Moore DD, Seidman J, et al., editors, Current Protocols in Molecular Biology, Hoboken, NJ, USA: John Wiley & Sons, Inc.

51. Li K (2008). The image stabilizer plugin for ImageJ. http://www.cs.cmu.edu/ kangli/code/Image_Stabilizer.html.

52. Lambert G (2014). Celltracker: a set of tools to track fluorescently tagged bacterial cells. http://github.com/guillambert/celltracker.

53. Neidhardt FC, Bloch PL, Smith DF (1974) Culture medium for enterobacteria. Journal of Bacteriology 119: 736–747.
